# Immunomodulatory effects of extracellular vesicles in glioblastoma

**DOI:** 10.3389/fcell.2022.996805

**Published:** 2022-11-16

**Authors:** Johannes Jun Wei Low, Siti Aishah Sulaiman, Nor Adzimah Johdi, Nadiah Abu

**Affiliations:** UKM Medical Molecular Biology Institute (UMBI), UKM Medical Centre, Universiti Kebangsaan Malaysia, Jalan Yaa’cob Latiff, Bandar Tun Razak, Kuala Lumpur, Malaysia

**Keywords:** glioma, exosome, immune cells, Tumor microenvironment, microvesicle (MV)

## Abstract

Glioblastoma (GB) is a type of brain cancer that can be considered aggressive. Glioblastoma treatment has significant challenges due to the immune privilege site of the brain and the presentation of an immunosuppressive tumor microenvironment. Extracellular vesicles (EVs) are cell-secreted nanosized vesicles that engage in intercellular communication *via* delivery of cargo that may cause downstream effects such as tumor progression and recipient cell modulation. Although the roles of extracellular vesicles in cancer progression are well documented, their immunomodulatory effects are less defined. Herein, we focus on glioblastoma and explain the immunomodulatory effects of extracellular vesicles secreted by both tumor and immune cells in detail. The tumor to immune cells, immune cells to the tumor, and intra-immune cells extracellular vesicles crosstalks are involved in various immunomodulatory effects. This includes the promotion of immunosuppressive phenotypes, apoptosis, and inactivation of immune cell subtypes, which affects the central nervous system and peripheral immune system response, aiding in its survival and progression in the brain.

## Introduction

Central nervous system (CNS) cancers comprise both primary and secondary CNS tumors in which the former are derived from CNS cells while the latter emerges from the spread of cancerous cells from the peripheral body to the brain (Lapointe et al., 2018; Achrol et al., 2019). The incidence rate for secondary CNS tumors is expected to be larger than primary CNS tumors (Davis et al., 2012), with the survival rate determined by the primary cancer location (Cagney et al., 2017). Nevertheless, the low incidence rate of primary CNS cancer is confounded by its high mortality rate (Miller et al., 2021). Gliomas, representing only 24.5% of all primary brain and CNS tumors, hold 80.9% of recorded malignant tumors, signifying the importance of this cancer as a modern-day killer (Ostrom et al., 2021). Currently, both glioblastoma, isocitrate dehydrogenase-wildtype (IDH-wildtype) and astrocytoma, IDH-mutant (previously glioblastoma, IDH-mutant) are classified as WHO Grade 4. Astrocytoma, IDH-mutant is a highly malignant astrocytic glioma with a low survival rate (6.8% survival rate post-diagnosis, median survival of 8 months) and portrayed mutations to either IDH1/2 (Louis et al., 2021; Ostrom et al., 2021). It was previously mentioned that glioblastoma emerges in two different types: 1) primary, in which it arises *de novo,* or 2) secondary, where it progresses from a lower grade astrocytoma (Ohgaki and Kleihues, 2013) but following the 2021 classification, the term “secondary glioblastoma” which commonly involves R132H mutation in IDH1 (92.7% of various brain tumors) was annulled (Balss et al., 2008; Louis et al., 2021). Glioblastoma (GB) arises *de novo* with a very fast progression rate (mean 6.3 months from the first symptom to definitive diagnosis) (Ohgaki and Kleihues, 2005; Ohgaki and Kleihues, 2007). Growth dynamics analysis conducted by Stensjoen et al. showed that untreated GB has a median specific growth rate of 1.4% every day, leading to an equivalent volume doubling time of 49.6 days, further showcasing the expedited growth of GB tumors (Stensjøen et al., 2015). GB tumors need to mediate an immunosuppressive microenvironment (Brown et al., 2018) through bidirectional communication with surrounding resident cells *via* several approaches including soluble factors, direct cell-cell contact, and extracellular vesicles (Broekman et al., 2018), where the latter is increasingly recognized as an important mediator of cell-cell communication (Gao et al., 2020).

The CNS is traditionally known as an “immune privileged” site due to several factors, including 1) the presence of a blood-brain barrier (BBB) that limits access to peripheral immune cells, 2) the lack of a lymphatic vessel serving the CNS which limits antigen trafficking and presentation in lymph nodes, 3) paucity of antigen-presenting cells (APC) in the CNS, 4) downregulation of major histocompatibility complex (MHC) expression in normal brain parenchyma leading to diluted T cell immune response, and 5) presence of anti-inflammatory modulators (Fabry et al., 2008; Brown et al., 2018). The CNS facilitates the entry and continued presence of immune cells to survey and respond against foreign entities such as tumors (Papadopoulos et al., 2020). Still, GB has long been considered a “cold” tumor with high intrinsic and adaptive resistance to immunotherapy (Jackson et al., 2019) due to intratumoral heterogeneity and lack of high-quality neoantigens, as well as severe dysregulation of immune cells favoring the immunosuppressive phenotype (Hao et al., 2002). Nevertheless, efforts have been taken to convert the “cold” GB phenotype to a more immunotherapy-susceptible “hot” phenotype (Tomaszewski et al., 2019). Glioma cells often engage with multiple glial cell types, including immune cells, to create an immunosuppressive tumor microenvironment (TME) *via* the secretion of pro-tumorigenic mediators (Quail and Joyce, 2017; Brown et al., 2018). Mechanisms relating to immune cells’ response in the GB microenvironment remain scarce and incompletely defined (Lim et al., 2018) although extracellular vesicles are found to be implicated in several instances stated further along in this review.

## Extracellular vesicles

EVs are small lipid-enclosed membrane vesicles secreted from virtually all kinds of cells into the extracellular spaces to engage in various cellular processes (Urabe et al., 2020). EVs can be classified based on their size: small EVs consist of particles <200 nm in diameter, medium EVs consist of particles between 200–400 nm in diameter, and large EVs consist of particles larger than 400 nm in diameter (Théry et al., 2018). Tumor cells-derived EVs play important roles in modulating the tumor microenvironment (TME) and promoting tumor progression *via* the transfer of tumor-specific molecules to recipient cells (Ricklefs et al., 2016). Effects of this intercellular communication include the establishment of a premetastatic niche, promoting angiogenesis, disruption to the peritoneum or BBB, chemotherapeutic drug resistance, and formation of heterogenous cancer-associated fibroblast (Urabe et al., 2020). Several studies have also described the variety of cargo carried within the vesicle including proteins and nucleic acids in both small and large EVs (Thakur et al., 2014; Hurwitz et al., 2016; García-Romero et al., 2017; Vagner et al., 2018). In essence, EVs allow biomolecules to be transported in a stable and protected format, allowing liquid biopsy utilizing patients’ blood. García-Romero et al. reported on the presence of glioma tumor-derived genomic DNA (gDNA) despite the presence of the BBB, denoting EV’s capability to bypass the anatomical restriction (García-Romero et al., 2017).

**FIGURE 1:**
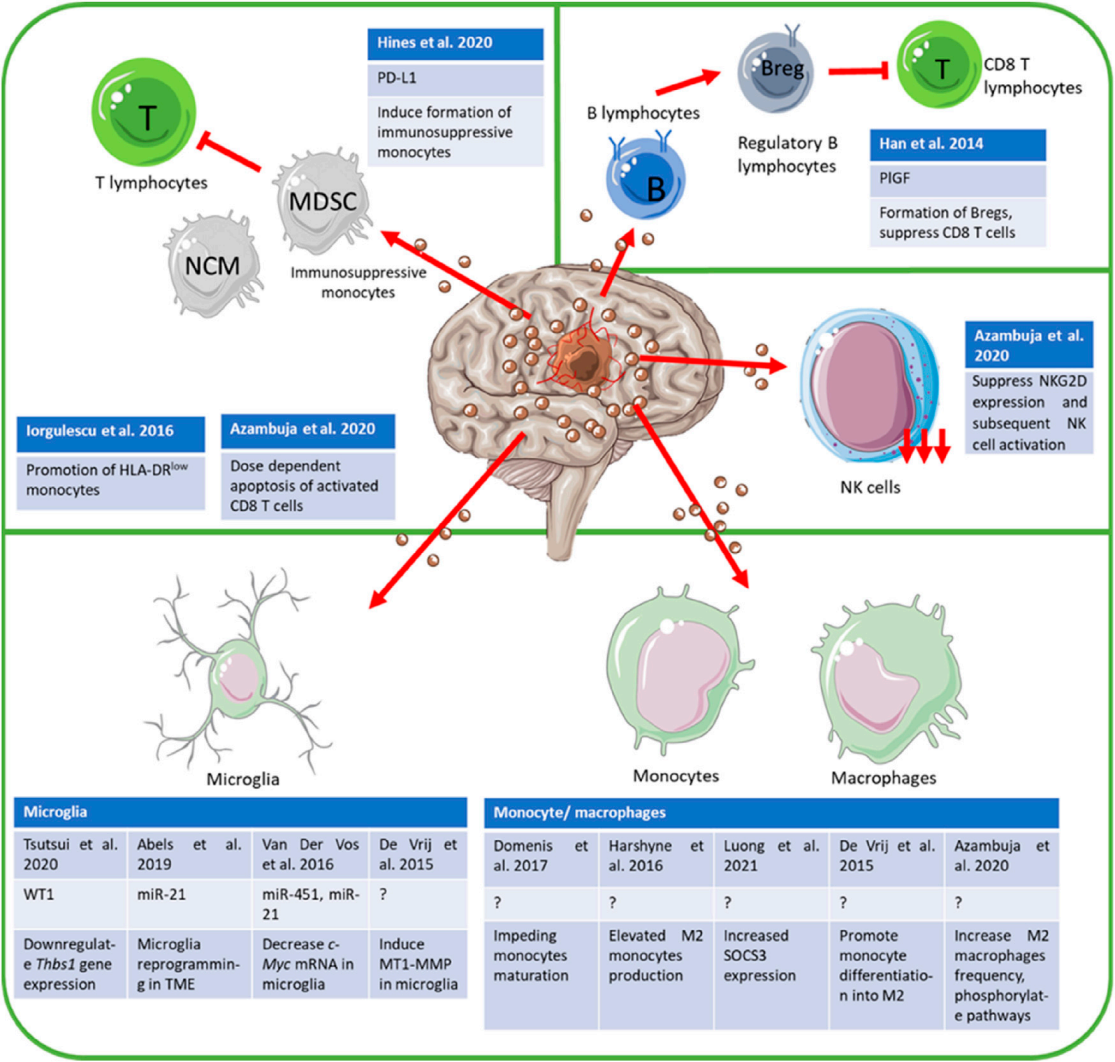
Schematic description of GB-derived EVs immunomodulation. GB performs immunosuppression by interfering with the expression of regulatory proteins *via* either expression of miRNAs or other proteins. Other than that, GM also impedes the maturation of immune cells such as monocytes, in addition to promoting the expression of immunosuppressive phenotypes in immune cells while downregulating the presence of immune-promoting phenotypes. Other immunosuppressive mechanisms employed included dose-dependent apoptosis of activated immune cells.

Biomolecules present in and on the extracellular vesicles are representative of parental cells and are often functional (Yekula et al., 2020). A study conducted by Kucharzewska et al. demonstrated that exosomes reflect the hypoxic status of glioma cells evidently through the cargo makeup of the exosome (Kucharzewska et al., 2013). Exosomal enzymes’ mRNA levels correspond to levels in parental cells, further denoting the presence of an elaborate cargo selection machinery in cells (Shao et al., 2015). Several mechanisms have been determined to influence cargo selection in EVs, notably the ADP-ribosylation factor 6 (ARF-6)-Exportin-5 axis, where ARF6-GTP interacts with Exportin-5 to deliver miRNA into tumor microvesicles (MVs) in an ARF6-GTP dependent manner (Clancy et al., 2019). Other than that, target proteins can also be sorted into EVs *via* ubiquitination, which is mainly mediated by endosomal sorting complexes required for transport (ESCRT) complex and partly by other proteins displaying Ubiquitin (Ub)-binding domains (Piper et al., 2014). Lipid sorting is not well understood, but Bissig and Gruenberg (Bissig and Gruenberg, 2013) summarized several important factors such as lipid-lipid/lipid-protein interaction, differing membrane biophysical traits, and metabolic enzyme turnover rate/distribution to be deeply involved with differing lipid populations in endosomes (Bissig and Gruenberg, 2013). Various other cargoes can also get sorted into exosomes *via* post-translational modifications (Moreno-Gonzalo et al., 2018). The schematic representation of GB-derived EVs immunosuppression is described in Figure 1.

EV is also instrumental in the metabolism modulation within the GB TME. Tumor-activated stromal cells (TASC), also known as cancer-associated fibroblasts have been determined to transfer mitochondria to primary GB cells *via* various mechanisms including EVs (Salaud et al., 2020). This led to increased glycolysis which translates to better GB proliferation (Salaud et al., 2020). Under favorable metabolic conditions, GB cells also utilize EVs as a vehicle to reduce intracellular miRNA content. This is evident when GB cells secrete exosomal miR-451, which is >40-fold more abundant in EVs compared to in cells (Van Der Vos et al., 2016). Although said EVs were absorbed by microglia, the increased release of exosomal miR-451 from GB cells coincides with a GB cell self-preservation mechanism when glucose is limited. Godlewski *et al.* (Godlewski et al., 2010) demonstrated when glucose is limited, miR-451 levels in the cell decline, leading to heightened CAB39 expression, activation of AMPK and consequently cell survival by reducing cell proliferation. GB-derived EVs are also capable of modulating other cells in the CNS to facilitate the TME. A study by Oushy *et al.* (Oushy et al., 2018) found that GB EV-treated normal human astrocytes (NHA) displayed enhanced migration ability and cytokine production, which are tumor-promoting phenotypes favored by GBM cells. The EV-mediated NHA drive to tumor-supporting phenotype was further elucidated by Hallal *et al.* (Hallal et al., 2019) when it was postulated that EV-treated NHA displayed a senescence-associated secretory profile (SASP) along with enhanced migration capabilities through enhanced podosome and gelatin matrix degradation. EV-mediated cell modulation in the GBM TME also occurs in immune cells, where tumor-associated macrophages (TAMs) are found to secrete *CHD7*-targeting microRNAs to glioma stem cells (GSCs) to trigger a proneural-to-mesenchymal transition (PMT) (Zhang et al., 2020). As PMT confers resistance to therapy, this contributes to worsening diagnosis in recurrent GBM (Fedele et al., 2019). Other than that, GSCs-derived EVs also induce the growth of brain endothelial cells (BEC), further enhancing their survival (Spinelli et al., 2018) which may be beneficial to the GBM TME. A study comparing glioma-derived human ECs (GhECs) and normal human ECs (NhECs) determined that GhEC-EV significantly induced LN229 GB cell line migration *in vitro via* MYO1C transfer to recipient cells (Tian et al., 2020). A study by Lucero *et al.* (Lucero et al., 2020) also discovered that GSC-derived EVs carry vasculature-associated miRNAs that reprogram brain EC to perform angiogenesis. EC is also vital for the formation of the blood-brain barrier (Kadry et al., 2020) yet a study by Treps *et al.* (Treps et al., 2016) discovered that Semaphorin3A expressed on GBM EVs’ surface causes increased vascular permeability *in vivo*, which can jeopardize the integrity of the BBB. GSC also secrete VEGF-A, a known angiogenic and permeability factor in the exosomal form to human BECs (Treps et al., 2017).

Immunotherapy-based strategies for GB have shown some preclinical successes that were not translated into Phase 3 clinical efficacies. (Yu and Quail, 2021). Therefore, looking into the TME might provide clues to targeting this issue. A paper by Ali *et al.* (Ali et al., 2021) showed that TME-targeting treatments including combination strategies led to a variety of post-treatment GB TME. GB cells have been shown to utilize extracellular vesicles (EVs) to circumvent immunotherapies and radiation therapy. For instance, U87 glioma cell line treated with bevacizumab has been shown to secrete out EVs containing bevacizumab on the surface (Simon et al., 2018) albeit with a slight influence on cell viability and proliferation in clinical dosages. Hypoxic glioma cells also secrete exosomal miR-301a that promotes resistance to radiotherapy (Yue et al., 2019). In terms of metabolism, the post-irradiation brain allows a tumor-tolerant microenvironment *via* metabolic shift with high production of energy carriers and low production of antioxidants (Gupta et al., 2020), which might also be translated into extracellular vesicles as well. This has been documented when GB cell lines of multiple subtypes exposed to either acute or chronic irradiation also exhibit metabolic changes that translate into the alteration of microvesicle cargo, ultimately modulating said vesicles’ paracrine signaling towards untreated glioma cells (Baulch et al., 2016). 

Radiation therapy and chemotherapy might also cause glioma cells to be more malignant. U87MG cells exposed to radiation also secrete more exosomes with enhanced cell migration capability due to an increased abundance of cell motility-related mRNA and proteins (Arscott et al., 2013). Pavlyukov *et al.* (Pavlyukov et al., 2018) also demonstrated apoptotic cell-derived EVs (apoEVs) from irradiated or chemo-treated glioma cells promote malignancy by phenotypic changes induced by splicing factor transfer to recipient glioma cells. A study by Ramakrishnan *et al.* (Ramakrishnan et al., 2020) also demonstrated that irradiation causes glioma cells to adopt a stem cell state by EV-mediated release of miR-603, causing resistance to ionizing radiation and DNA alkylating drugs. In terms of chemotherapy, temozolomide administered to glioma stem cells also induce secretion of GSC-derived EVs that are enriched with cell adhesion proteins which might aid in tumor progression (Andre-Gregoire et al., 2018). TMZ-induced EV cargo change is also reported by Garnier *et al.* (Garnier et al., 2018) when they determined that EVs secreted by GB cell lines contained TMZ resistance transcripts. A comprehensive study by Cuperlovic-Culf *et al.* (Cuperlovic-Culf et al., 2020) on GB cell line-derived EVs also showed that metabolome cargoes are implicated in immune response and metabolism amid being varied depending on the cell line. 

### Crosstalk between extracellular vesicles and T cells

T cells’ secretion of EVs is documented to be upregulated post T cell receptor (TCR) triggering, which possibly mediates surface TCR/CD3-mediated cell homing (Blanchard et al., 2002). Other than that, overactive T cells are also capable of secreting Fas ligand (FasL) and Apo2 ligand (APO2L) *via* EV release, suggesting EV-mediated autocrine or paracrine immune regulation (Monleón et al., 2001). Delivery of specific cargo to recipient cells is also evident with mechanisms including sequence motifs-dependent microRNA localization into exosomes (Villarroya-Beltri et al., 2013) and monophosphorylation-dependent FasL sorting into secreted lysosomes (Zuccato et al., 2007). Nevertheless, death ligands need to be bound on the membrane to crosslink efficiently with their corresponding death receptors (Anel et al., 2019). In general, T cells EVs deliver cargoes such as enzymes, transmembrane proteins, members of the immunoglobulins (Ig) superfamily, and MHC molecules to target cells such as APCs (Choudhuri et al., 2014; Mittelbrunn et al., 2011; Nolte-'t Hoen et al., 2004) and B cells (Yang et al., 2019). Multiple responses in target cells were recorded, including activation-induced cell death (AICD) where microvesicles containing FasL and APO2L were released shortly before cell apoptosis (Martínez-Lorenzo et al., 1999). Positive and negative regulation of T cell responses were also recorded. T cell-derived EVs carry surface receptors and molecules to APCs, causing transcellular signaling and modulation of APCs (Choudhuri et al., 2014; Nolte-'t Hoen et al., 2004). T cell-derived exosomes are also capable of transporting miRNA to APCs, causing changes to recipient cells’ gene expressions (Mittelbrunn et al., 2011).

Tregs, owning to their immunosuppressive traits, also secrete exosomes that perform similar functions. Immunosuppression mediated by Tregs involves several different mechanisms, including adenosine (Ado) mediated immunomodulation (Smyth et al., 2013; Schuler et al., 2014), cyclooxygenase-2 (Cox-2) mediated regulation of interferon-gamma (IFNγ) secretion in T effector cells (Okoye et al., 2014), target cell cycle arrest and apoptosis, conversion of T cells into Tregs (Aiello et al., 2017), induction of tolerogenic dendritic cells (DCs) (Tung et al., 2018), and increased resistance to apoptosis by Tregs cells (Czystowska et al., 2010). GB cell line-derived exosomes contain inhibitory proteins such as CD39, FasL, CTLA-4, TRAIL, and CD73 that attenuate the normal function of all immune cells (Azambuja et al., 2020). GB has been documented to release PD-L1^+^ EVs which inhibit T cell proliferation *via* myeloid-derived suppressor cells (MDSC) and nonclassical monocytes (NCM) instead of direct T cell inhibition (Himes et al., 2020). The mechanism of action is in contrast to findings by Ricklefs et al. who argue GB-derived EVs block TCR-mediated T cell activation (Ricklefs et al., 2018), although Hines et al. do point out the possible discrepancy with T cell stimulation employed by both researchers causing contrasting results regarding GB EVs role (Himes et al., 2020). GB-derived exosomes have limited ability to activate CD8^+^ T lymphocytes (Iorgulescu et al., 2016) yet it has been reported that the expression of CD86 on glioma cell lines can be bound competitively by cytotoxic T-lymphocyte-associated protein 4 (CTLA-4), leading to possible T cell immunosuppression (Walker and Sansom, 2011). Therefore, more research is needed to elucidate the GB-derived EV effect on T cells. The crosstalk between EVs and T cells, and other immune cells are tabulated in Table 1.

**TABLE 1:** Examples of immune cells -glioblastoma EV crosstalk.

Donor cells	Types of EV	Participating molecules	Receptor cells	Role/Function	References
Tregs	Exosomes	miR-150–5p, miR-142–3p	Dendritic cells	miR-150–5p, miR-142–3p mediated induction of tolerogenic phenotype in DCs, leading to increased IL-10 and decreased IL-6 production following LPS stimulation	Tung et al. (2018)
Foxp3^+^ T regulatory (Treg) cells	Exosome	Various miRNAs	Effector T cell	Treg-mediated immunosuppression *via* secretion of miRNA-containing exosomes	Okoye et al. (2014)
Tregs	EV	miR-150–5p and miR-142–3p	Dendritic cells	GBM-derived EVs release PD-L1+ EVs and induce the formation of MDSC and PD-1+ NCM	Tung et al. (2018)
GBM	EV	PD-L1	T cells, monocytes, dendritic cells	GBM-derived EVs release PD-L1+ EVs and induce the formation of MDSC and PD-1+ NCM	Himes et al. (2020)
Glioma	Exosomes	-	Peripheral monocytes, CD8^+^ T lymphocytes	Promotion of immunosuppressive HLA-DR^low^ monocytic phenotypes; glioma-derived exosomes lacked antigen-presentation machinery and surface co-modulatory molecules	Iorgulescu et al. (2016)
Glioma	EVs, particularly exosomes	Wilms tumor-1 (WT-1)	Microglia	Glioma-derived EVs promote tumor progression by affecting microglial gene expression and promoting microglial recruitment and angiogenesis.WT1 in EVs downregulates microglial Thbs1 gene expression	Tsutsui et al. (2020)
Glioma cells	EV	miR-21	Microglia	EV-derived miR-21 is functionally transferred from glioma to microglia through EVs *in vivo*, mediating reprogramming of microglia in the tumor microenvironment through increased post Btg2 downregulation	Abels et al. (2019)
Hypoxia glioma	Exosomes	miR-10a, miR-21	MDSC	MDSC expansion and activation *via* RORA and PTEN silencing	Guo et al. (2018)
Glioma stem cell	Exosomes	-	PBMCs, CD14^+^ monocyte	GSC-derived exosomes and exosomes from GBM peripheral blood suppress the peripheral T-cell immune response by acting on monocyte maturation rather than on direct interaction with T cells, skewing them toward a monocytic-MDSC tumor-supportive phenotype	Domenis et al. (2017)
GB	Serum exosomes and cytokines	-	Normal monocytes	M2-like monocytes expressing CD14^+^ and CD163 are elevated in GB patient blood, indicating Th2 bias	Harshyne et al. (2016)
GB	EV	miR-451/miR-21	Microglia	Microglia avidly took up GB-EVs, causing increased proliferation and shifting their cytokine profile toward immune suppression	Van Der Vos et al. (2016)
GB, GSC	EV	GSC: CSPG4, PTGFRN and DIP2B	Peripheral blood-derived monocytes, Microglia	GB EVs promote differentiation of peripheral blood-derived monocytes into M2 macrophages, induced changes to cell surface protein expression, cytokine secretion and increased phagocytic capacity; induced microglia to be tumor supportive	De Vrij et al. (2015)
		GB: α2M, EDIL3, and HBB.			
		Shared: CSPG4, α2M, MFGE8 (lactadherin), EGFR and different types of integrins			
Glioma	EV	-	Monocytes	Glioma EV	Luong et al. (2021)
				• Increased SOCS3 expression in monocytes	
				• Decreased MHCII, CD80 expression	
				• Increased PDL1, Ly6C expression	
				• Increased suppressive cytokines and immune mediators’ expression (IL-10, TGFb, arginase, iNOS)	
				• Production of monocytes, causing decreased activated CD4^+^ T cell proliferation	
Patients-derived glioma tissues	Exosomes	Placental growth factor (PlGF)	B cells	Glioma-derived exosomes containing PlGF induce differentiation of B cells into glioma-specific regulatory B cells, causing suppression of glioma-specific CD8^+^ T cells	Han et al. (2014)

### Effects of extracellular vesicles on myeloid cells

Given myeloid cells’ extensive presence in the brain, gliomas’ interaction with them is considerably more frequent, where up to 30% of total glioma/glioblastoma mass is comprised of myeloid cells (Arcuri et al., 2017). Gliomas utilize this concentration of myeloid cells to their advantage by secreting EVs capable of modulating microglia. The WT-1 protein is found secreted in glioma-derived EVs and downregulates *thrombospondin-1* (*Thbs1*) in microglia, subsequently promoting angiogenesis which is vital in glioma progression (Wagner et al., 2014; Tsutsui et al., 2020). Other than that, microglia proliferation is also promoted *via* downregulation of BTG anti-proliferation factor 2 (*Btg2*) expression in microglia post-delivery of miR-21 through EVs (Abels et al., 2019).

Glioma cells also exhibit EV-mediated malignancy under hypoxic conditions. Under hypoxic conditions, glioma cells secrete exosomes containing TERF2 interacting protein (TERF2IP) targeting-miR-1246, causing activation and inhibition of STAT3 and Nuclear factor-κB (NF-κB) signaling pathways respectively. This leads to the polarization of macrophages from M1 to immunosuppressive M2 phenotype (Qian et al., 2020). Glioma cells under hypoxic conditions have also been found to secrete exosomes containing miR-10a and miR-21 to MDSCs, causing its expansion and activation (Guo et al., 2018), which further exacerbates cancer progression *via* inhibition of immune cell functions, Tregs expansion and promotion of immunosuppressive regulatory B cells (Bregs) (Mi et al., 2020). Other than that, GB affects T-cell immune response through the modulation of monocytes. Systemic T cell suppression *via* glioma stem cells (GSCs) derived exosomes occurs with internalization by CD14^+^ monocyte which causes stunted maturation and formation of monocytic MDSCs, subsequently disrupting CD3^+^ and CD4^+^ T cell activation (Domenis et al., 2017). GB EVs also induces the proliferation of NCMs and MDSCs, eventually inhibiting T cell proliferation (Himes et al., 2020).

GB is also able to influence the peripheral immune environment *via* exosomes as M2-like monocytes expressing CD163 and CD14 are highly expressed in the GB patients’ peripheral blood, representing a Th2 bias (Harshyne et al., 2016). GB tumors also secrete miR-451 and miR-21 in EVs, which when internalized by microglia, cause an increase in microglia proliferation and a cytokine shift favoring immune suppression (Van Der Vos et al., 2016). GB EVs also contain leukocyte migration and focal adhesion-specific proteins that skewed peripheral monocytes’ differentiation towards M2 macrophages in addition to modification to macrophages’ cell surface protein expression, cytokine secretion, and phagocytic effect (De Vrij et al., 2015). De Vrij et al. also mentioned human microglia exhibit high expression of membrane-type 1-matrix metalloproteinase (MT1-MMP) post-incubation with GB EVs, which supports tumor growth, denoting the tumor proliferation aspect of cancer-associated immune cells in addition to their immunoregulatory role (De Vrij et al., 2015). Luong *et al.* (Luong et al., 2021) also determined that glioma-derived EVs perform several pro-tumorigenic functions in monocytes such as upregulated expression of suppressive cytokines, proteins, PD-L1 and lymphocyte antigen six complex (Ly6C), downregulation of proinflammatory cytokines, MHC II and costimulatory CD80 expression as well as the conversion of monocytes into suppressive cells involved in inhibition of activated CD4^+^ T cells (Luong et al., 2021). IFN-γ stimulation of GB cells also causes superinduction of GB-derived, immunosuppressive IDO-1 and PD-L1 expressing EVs, which led to immunosuppression of monocytes on top of differentiation of monocytes into immunosuppressive MDSCs and NCMs (Jung et al., 2022).

### Effects of extracellular vesicles on NK cells

NK cells are also known to secrete extracellular vesicles containing a variety of biomolecules, including membrane and extracellular matrix (ECM) proteins (tetraspanins, integrins), death receptor ligands, cytolytic enzymes, and miRNA that translate into cytotoxic effect in cancer cells such as apoptosis while avoiding damage to normal PBMCs (Farcas and Inngjerdingen, 2020). Other than that, NK extracellular vesicles (NKEVs) cause immunomodulation in PBMCs including higher expression of HLA-DR and costimulatory molecules on monocytes, CD25 on T cells, and CD56 on NK cells, which translates into both pro-inflammation and anti-inflammation spectrum of the immune response (Federici et al., 2020). NK exosomes also contain FasL, Natural Killer Group 2D (NKG2D), and perforin molecules, which perform an individualistic function: perforin mediates tumor and activated immune cell death in a time and dose-dependent manner, while Fas is suggested to be involved in lymphocyte homeostasis regulation (Lugini et al., 2012). NKG2D, typically known as an activating receptor by cytolytic lymphoid cells (Pende et al., 2001), typically can induce a cytolytic effect without more specific natural cytotoxicity NK receptors, yet their functional role in exosomes is not known. Activated NKEVs induced dose-dependent caspase-mediated apoptosis in neuroblastoma *via* functional perforin, granulysin, and granzymes A and B mediation of the caspase pathway (Jong et al., 2017) which possibly translates into a similar result in GB. As per other immune cells, tumor-derived MVs secreted under hypoxic conditions are more potent in impairing NK cytotoxicity and cell function as compared to normoxic equivalent (Berchem et al., 2016) due to the delivery of CD107a targeting-miR23a and TGF-β (Alter et al., 2004; Viel et al., 2016). NK EVs are also multifaceted in immunomodulation. Azambuja et al. mentioned that GB-derived exosomes suppress NK cell activation by suppressing NKG2D expression levels (Azambuja et al., 2020). In contrast to this, NK cells also secrete cytotoxic EVs under the influence of pro-inflammatory cytokines (Enomoto et al., 2021) with IL-15 and IL-21 both playing significant roles in NK cell activation (Carson et al., 1994; Skak et al., 2008). All in all, studies have shown that NK cell activity can be modulated by tumor-derived EVs, including those of GB origin, with the effect dependent on external stimuli such as the balance between immune promoting- and inhibiting-signals, further confirming the notion that NK cell function is niche dependent.

### Effects of extracellular vesicles on B cells

B cell’s secretion of EVs has been documented with the release of MHC II-containing exosomes (Raposo et al., 1996), which it was suggested to involve in antigen presentation (Lindenbergh and Stoorvogel, 2018). Muntasell et al. reported on upregulated B cell-derived exosome carrying antigenic-peptide MHC II (pMHC II) by the antigen-specific CD4^+^ T cell, which can poise as a positive modulator for ongoing immune response and maintenance of antigenic memory in T cells (Muntasell et al., 2007). Given so, B cells exosomes are also implicated in anti-inflammatory responses such as the possible transfer of membrane-tethered CD73 to Tregs in the peripheral causing an increase in anti-inflammatory adenosine secretion (Schuler et al., 2014). A more recent paper by Zhang et al. also demonstrated that CD19^+^ EVs secreted from B cells induce hydrolysis of adenosine triphosphate (ATP) to adenosine *via* incorporated CD39 and CD73 action, thus inhibiting CD8^+^ T cell proliferation and subsequent reduction in chemotherapy efficacy (Zhang et al., 2019). Although both studies do not employ GB as their model, the GB tumor microenvironment is known to be hypoxic (Brown et al., 2018; Tomaszewski et al., 2019), and CD73 is found to be upregulated in both CNS (Kulesskaya et al., 2013) and hypoxic conditions (Li et al., 2006). B cells in the CNS might also participate in the upregulation of adenosine, especially when Zhang et al. also mentioned that hypoxia-inducible factor-1α (HIF-1α) mediated Rab27a expression causes heightened EV secretion by B cells (Zhang et al., 2019). Glioma cells also participated in B cell modulation. Glioma cells secrete placental growth factor (PlGF) in exosomal form, which in contact with naïve B cells can induce their differentiation to Bregs (Han et al., 2014). Induced Bregs suppress granzyme B and perforin secretion by the glioma-specific CD8^+^ T cell, denoting specificity in B cell action against glioma. However, relevant publications regarding B cells exosome against glioma are still lacking.

## Conclusion

In conclusion, EVs play vital roles in both faces of the immunopathological aspects of GB. EVs typically reflect the physiological state of the donor cell and perform specific effector functions in recipient cells, thus modulating the recipient cells to have an abnormal phenotype. This plays well into the induction of the TME, which in the GB context involves the relationship between GB tumors and neighboring cells, promoting immunosuppression and proliferation within the brain. How the common cell adjusts its metabolic requirement to fit into the objective of the tumor microenvironment is an interesting avenue to research, especially with the usage of EVs, which can be a vehicle for both paracrine and endocrine signaling in the first place. More thorough EV research across all facets of cell biochemistry is needed to synthesize, elucidate, and magnify possible influential pathways in the cell, where such results can be translated into more optimized and effective screening and treatment strategies in the future.

## References

[B1] Abels E. R. Maas S. L. N. Nieland L. Wei Z. Cheah P. S. Tai E. (2019). Glioblastoma-associated microglia reprogramming is mediated by functional transfer of extracellular miR-21. Cell Rep. 28, 3105–3119. 10.1016/j.celrep.2019.08.036 31533034 PMC6817978

[B2] Achrol A. S. Rennert R. C. Anders C. Soffietti R. Ahluwalia M. S. Nayak L. (2019). Brain metastases. Nat. Rev. Dis. Prim. 5, 5. 10.1038/s41572-018-0055-y 30655533

[B3] Aiello S. Rocchetta F. Longaretti L. Faravelli S. Todeschini M. Cassis L. (2017). Extracellular vesicles derived from T regulatory cells suppress T cell proliferation and prolong allograft survival. Sci. Rep. 7, 11518–11519. 10.1038/s41598-017-08617-3 28912528 PMC5599553

[B4] Ali S. Borin T. F. Piranlioglu R. Ara R. Lebedyeva I. Angara K. (2021). Changes in the tumor microenvironment and outcome for TME-targeting therapy in glioblastoma: A pilot study. PLoS One 16, e0246646. 10.1371/journal.pone.0246646 33544755 PMC7864405

[B5] Alter G. Malenfant J. M. Altfeld M. (2004). CD107a as a functional marker for the identification of natural killer cell activity. J. Immunol. Methods 294, 15–22. 10.1016/j.jim.2004.08.008 15604012

[B6] Andre-Gregoire G. Bidere N. Gavard J. (2018). Temozolomide affects extracellular vesicles released by glioblastoma cells. Biochimie 155, 11–15. 10.1016/j.biochi.2018.02.007 29454008

[B7] Anel A. Gallego-Lleyda A. de Miguel D. Naval J. Martínez-Lostao L. (2019). Role of exosomes in the regulation of T-cell mediated immune responses and in autoimmune disease. Cells 8, 154. 10.3390/cells8020154 30759880 PMC6406439

[B8] Arcuri C. Fioretti B. Bianchi R. Mecca C. Tubaro C. Beccari T. (2017). Microglia-glioma cross-talk: A two way approach to new strategies against glioma. Front. Biosci. 22, 268–309. 10.2741/4486 27814616

[B9] Arscott W. T. Tandle A. T. Zhao S. Shabason J. E. Gordon I. K. Schlaff C. D. (2013). Ionizing radiation and glioblastoma exosomes: implications in tumor biology and cell migration. Transl. Oncol. 6, 638–648. 10.1593/tlo.13640 24466366 PMC3890698

[B10] Azambuja J. H. Ludwig N. Yerneni S. Rao A. Braganhol E. Whiteside T. L. (2020). Molecular profiles and immunomodulatory activities of glioblastoma-derived exosomes. Neurooncol. Adv. 2, vdaa056–11. 10.1093/noajnl/vdaa056 32642708 PMC7262743

[B11] Balss J. Meyer J. Mueller W. Korshunov A. Hartmann C. von Deimling A. (2008). Analysis of the IDH1 codon 132 mutation in brain tumors. Acta Neuropathol. 116, 597–602. 10.1007/s00401-008-0455-2 18985363

[B12] Baulch J. E. Geidzinski E. Tran K. K. Yu L. Zhou Y. H. Limoli C. L. (2016). Irradiation of primary human gliomas triggers dynamic and aggressive survival responses involving microvesicle signaling. Environ. Mol. Mutagen. 57, 405–415. 10.1002/em.21988 26602180

[B13] Berchem G. Noman M. Z. Bosseler M. Paggetti J. Baconnais S. Le cam E. (2016). Hypoxic tumor-derived microvesicles negatively regulate NK cell function by a mechanism involving TGF-β and miR23a transfer. OncoImmunology 5, e1062968. 10.1080/2162402X.2015.1062968 27141372 PMC4839360

[B14] Bissig C. Gruenberg J. (2013). Lipid sorting and multivesicular endosome biogenesis. Cold Spring Harb. Perspect. Biol. 5, a016816. 10.1101/cshperspect.a016816 24086044 PMC3783046

[B15] Blanchard N. Lankar D. Faure F. Regnault A. Dumont C. Raposo G. (2002). TCR activation of human T cells induces the production of exosomes bearing the TCR/CD3/zeta complex. J. Immunol. 168, 3235–3241. 10.4049/jimmunol.168.7.3235 11907077

[B16] Broekman M. L. Maas S. L. N. N. Abels E. R. Mempel T. R. Krichevsky A. M. Breakefield X. O. (2018). Multidimensional communication in the microenvirons of glioblastoma. Nat. Rev. Neurol. 14, 482–495. 10.1038/s41582-018-0025-8 29985475 PMC6425928

[B17] Brown N. F. Carter T. J. Ottaviani D. Mulholland P. (2018). Harnessing the immune system in glioblastoma. Br. J. Cancer 119, 1171–1181. 10.1038/s41416-018-0258-8 30393372 PMC6251037

[B18] Cagney D. N. Martin A. M. Catalano P. J. Redig A. J. Lin N. U. Lee E. Q. (2017). Incidence and prognosis of patients with brain metastases at diagnosis of systemic malignancy: A population-based study. Neuro. Oncol. 19, 1511–1521. 10.1093/neuonc/nox077 28444227 PMC5737512

[B19] Carson W. E. Giri J. G. Lindemann M. J. Linett M. L. Ahdieh M. Paxton R. (1994). Interleukin (IL) 15 is a novel cytokine that activates human natural killer cells via components of the IL-2 receptor. J. Exp. Med. 180, 1395–1403. 10.1084/jem.180.4.1395 7523571 PMC2191697

[B20] Choudhuri K. Llodrá J. Roth E. W. Tsai J. Gordo S. Wucherpfennig K. W. (2014). Polarized release of T-cell-receptor-enriched microvesicles at the immunological synapse. Nature 507, 118–123. 10.1038/nature12951 24487619 PMC3949170

[B21] Clancy J. W. Zhang Y. Sheehan C. D’Souza-Schorey C. (2019). An ARF6–Exportin-5 axis delivers pre-miRNA cargo to tumour microvesicles. Nat. Cell Biol. 21, 856–866. 10.1038/s41556-019-0345-y 31235936 PMC6697424

[B22] Cuperlovic-Culf M. Khieu N. H. Surendra A. Hewitt M. Charlebois C. Sandhu J. K. (2020). Analysis and simulation of glioblastoma cell lines-derived extracellular vesicles metabolome. Metabolites 10, E88. 10.3390/metabo10030088 PMC7142482 32131411

[B23] Czystowska M. Strauss L. Bergmann C. Szajnik M. Rabinowich H. Whiteside T. L. (2010). Reciprocal granzyme/perforin-mediated death of human regulatory and responder T cells is regulated by interleukin-2 (IL-2). J. Mol. Med. 88, 577–588. 10.1007/s00109-010-0602-9 20225066 PMC3777742

[B24] Davis F. G. Dolecek T. A. McCarthy B. J. Villano J. L. (2012). Toward determining the lifetime occurrence of metastatic brain tumors estimated from 2007 United States cancer incidence data. Neuro. Oncol. 14, 1171–1177. 10.1093/neuonc/nos152 22898372 PMC3424213

[B25] De Vrij J. Niek Maas S. L. Kwappenberg K. M. C. Schnoor R. Kleijn A. Dekker L. (2015). Glioblastoma-derived extracellular vesicles modify the phenotype of monocytic cells. Int. J. Cancer 137, 1630–1642. 10.1002/ijc.29521 25802036

[B26] Domenis R. Cesselli D. Toffoletto B. Bourkoula E. Caponnetto F. Manini I. (2017). Systemic T cells immunosuppression of glioma stem cell-derived exosomes is mediated by monocytic myeloid-derived suppressor cells. PLoS ONE 12, e0169932. 10.1371/journal.pone.0169932 28107450 PMC5249124

[B27] Enomoto Y. Li P. Jenkins L. M. Anastasakis D. Lyons G. C. Hafner M. (2021). Cytokine-enhanced cytolytic activity of exosomes from NK Cells. Cancer Gene Ther. 3, 734–749. 10.1038/s41417-021-00352-2 PMC9209332 34316033

[B28] Fabry Z. Schreiber H. A. Harris M. G. Sandor M. (2008). Sensing the microenvironment of the central nervous system: immune cells in the central nervous system and their pharmacological manipulation. Curr. Opin. Pharmacol. 8, 496–507. 10.1016/j.coph.2008.07.009 18691672 PMC2614337

[B29] Farcas M. Inngjerdingen M. (2020). Natural killer cell–derived extracellular vesicles in cancer therapy. Scand. J. Immunol. 92, 129388–7. 10.1111/sji.12938 32697853

[B30] Fedele M. Cerchia L. Pegoraro S. Sgarra R. Manfioletti G. (2019). Proneural-mesenchymal transition: Phenotypic plasticity to acquire multitherapy resistance in glioblastoma. Int. J. Mol. Sci. 20, E2746. 10.3390/ijms20112746 PMC6600373 31167470

[B31] Federici C. Shahaj E. Cecchetti S. Camerini S. Casella M. Iessi E. (2020). Natural-killer-derived extracellular vesicles: Immune sensors and interactors. Front. Immunol. 11, 262–324. 10.3389/fimmu.2020.00262 32231660 PMC7082405

[B32] Gao X. Zhang Z. Mashimo T. Shen B. Nyagilo J. Wang H. (2020). Gliomas interact with non-glioma brain cells via extracellular vesicles. Cell Rep. 30, 2489–2500. 10.1016/j.celrep.2020.01.089 32101730

[B33] García-Romero N. Carrión-Navarro J. Esteban-Rubio S. Lázaro-Ibáñez E. Peris-Celda M. Alonso M. M. (2017). DNA sequences within glioma-derived extracellular vesicles can cross the intact blood-brain barrier and be detected in peripheral blood of patients. Oncotarget 8, 1416–1428. 10.18632/oncotarget.13635 27902458 PMC5352065

[B34] Garnier D. Meehan B. Kislinger T. Daniel P. Sinha A. Abdulkarim B. (2018). Divergent evolution of temozolomide resistance in glioblastoma stem cells is reflected in extracellular vesicles and coupled with radiosensitization. Neuro. Oncol. 20, 236–248. 10.1093/neuonc/nox142 29016925 PMC5777501

[B35] Godlewski J. Nowicki M. O. Bronisz A. Nuovo G. Palatini J. De Lay M. (2010). MicroRNA-451 regulates LKB1/AMPK signaling and allows adaptation to metabolic stress in glioma cells. Mol. Cell 37, 620–632. 10.1016/j.molcel.2010.02.018 20227367 PMC3125113

[B36] Guo X. Qiu W. Liu Q. Qian M. Wang S. Zhang Z. (2018). Immunosuppressive effects of hypoxia-induced glioma exosomes through myeloid-derived suppressor cells via the miR-10a/Rora and miR-21/Pten Pathways. Oncogene 37, 4239–4259. 10.1038/s41388-018-0261-9 29713056

[B37] Gupta K. Vuckovic I. Zhang S. Xiong Y. Carlson B. L. Jacobs J. (2020). Radiation induced metabolic alterations associate with tumor aggressiveness and poor outcome in glioblastoma. Front. Oncol. 10, 535. 10.3389/fonc.2020.00535 32432031 PMC7214818

[B38] Hallal S. Mallawaaratchy D. M. Wei H. Ebrahimkhani S. Stringer B. W. Day B. W. (2019). Extracellular vesicles released by glioblastoma cells stimulate normal astrocytes to acquire a tumor-supportive phenotype via p53 and MYC signaling pathways. Mol. Neurobiol. 56, 4566–4581. 10.1007/s12035-018-1385-1 30353492 PMC6505517

[B39] Han S. Feng S. Ren M. Ma E. Wang X. Xu L. (2014). Glioma cell-derived placental growth factor induces regulatory B cells. Int. J. Biochem. Cell Biol. 57, 63–68. 10.1016/j.biocel.2014.10.005 25450457

[B40] Hao C. Parney I. F. Roa W. H. Turner J. Petruk K. C. Ramsay D. A. (2002). Cytokine and cytokine receptor mRNA expression in human glioblastomas: Evidence of Th1, Th2 and Th3 cytokine dysregulation. Acta Neuropathol. 103, 171–178. 10.1007/s004010100448 11810184

[B41] Harshyne L. A. Nasca B. J. Kenyon L. C. Andrews D. W. Hooper D. C. (2016). Serum exosomes and cytokines promote a T-helper cell type 2 environment in the peripheral blood of glioblastoma patients. Neuro. Oncol. 18, 206–215. 10.1093/neuonc/nov107 26180083 PMC4724173

[B42] Himes B. T. Peterson T. E. de Mooij T. Garcia L. M. C. Jung M. Y. Uhm S. (2020). The role of extracellular vesicles and PD-L1 in glioblastoma-mediated immunosuppressive monocyte induction. Neuro. Oncol. 22, 967–978. 10.1093/neuonc/noaa029 32080744 PMC7339906

[B43] Hurwitz S. N. Rider M. A. Bundy J. L. Liu X. Singh R. K. Meckes D. G. (2016). Proteomic profiling of NCI-60 extracellular vesicles uncovers common protein cargo and cancer type-specific biomarkers. Oncotarget 7, 86999–87015. 10.18632/oncotarget.13569 27894104 PMC5341331

[B44] Iorgulescu J. B. Ivan M. E. Safaee M. Parsa A. T. (2016). The limited capacity of malignant glioma-derived exosomes to suppress peripheral immune effectors. J. Neuroimmunol. 290, 103–108. 10.1016/j.jneuroim.2015.11.025 26711578

[B45] Jackson C. M. Choi J. Lim M. (2019). Mechanisms of immunotherapy resistance: Lessons from glioblastoma. Nat. Immunol. 20, 1100–1109. 10.1038/s41590-019-0433-y 31358997

[B46] Jong A. Y. Wu C. H. Li J. Sun J. Fabbri M. Wayne A. S. (2017). Large-scale isolation and cytotoxicity of extracellular vesicles derived from activated human natural killer cells. J. Extracell. Vesicles 6, 1294368. 10.1080/20013078.2017.1294368 28326171 PMC5345580

[B47] Jung M. Y. Aibaidula A. Brown D. A. Himes B. T. Cumba Garcia L. M. Parney I. F. (2022). Superinduction of immunosuppressive glioblastoma extracellular vesicles by IFN-gamma through PD-L1 and IDO1. Neurooncol. Adv. 4, vdac017. 10.1093/noajnl/vdac017 35990703 PMC9389426

[B48] Kadry H. Noorani B. Cucullo L. (2020). A blood-brain barrier overview on structure, function, impairment, and biomarkers of integrity. Fluids Barriers CNS 17, 69. 10.1186/s12987-020-00230-3 33208141 PMC7672931

[B49] Kucharzewska P. Christianson H. C. Welch J. E. Svensson K. J. Fredlund E. Ringnér M. (2013). Exosomes reflect the hypoxic status of glioma cells and mediate hypoxia-dependent activation of vascular cells during tumor development. Proc. Natl. Acad. Sci. U. S. A. 110, 7312–7317. 10.1073/pnas.1220998110 23589885 PMC3645587

[B50] Kulesskaya N. Võikar V. Peltola M. Yegutkin G. G. Salmi M. Jalkanen S. (2013). CD73 is a major regulator of adenosinergic signalling in mouse brain. PLoS ONE 8, e66896. 10.1371/journal.pone.0066896 23776700 PMC3680420

[B51] Lapointe S. Perry A. Butowski N. A. (2018). Primary brain tumours in adults. Lancet 392, 432–446. 10.1016/S0140-6736(18)30990-5 30060998

[B52] Li X. Zhou T. Zhi X. Zhao F. Yin L. Zhou P. (2006). Effect of hypoxia/reoxygenation on CD73 (ecto-5′-nucleotidase) in mouse microvessel endothelial cell lines. Microvasc. Res. 72, 48–53. 10.1016/j.mvr.2006.04.005 16828810

[B53] Lim M. Xia Y. Bettegowda C. Weller M. (2018). Current state of immunotherapy for glioblastoma. Nat. Rev. Clin. Oncol. 15, 422–442. 10.1038/s41571-018-0003-5 29643471

[B54] Lindenbergh M. F. S. Stoorvogel W. (2018). Antigen presentation by extracellular vesicles from professional antigen-presenting cells. Annu. Rev. Immunol. 36, 435–459. 10.1146/annurev-immunol-041015-055700 29400984

[B55] Louis D. N. Perry A. Wesseling P. Brat D. J. Cree I. A. Figarella-Branger D. (2021). The 2021 WHO classification of tumors of the central nervous system: a summary. Neuro. Oncol. 23, 1231–1251. 10.1093/neuonc/noab106 34185076 PMC8328013

[B56] Lucero R. Zappulli V. Sammarco A. Murillo O. D. Cheah P. S. Srinivasan S. (2020). Glioma-derived miRNA-containing extracellular vesicles induce angiogenesis by reprogramming brain endothelial cells. Cell Rep. 30, 2065–2074. 10.1016/j.celrep.2020.01.073 32075753 PMC7148092

[B57] Lugini L. Cecchetti S. Huber V. Luciani F. Macchia G. Spadaro F. (2012). Immune surveillance properties of human NK cell-derived exosomes. J. Immunol. 189, 2833–2842. 10.4049/jimmunol.1101988 22904309

[B58] Luong N. Lenz J. A. Modiano J. F. Olson J. K. (2021). Extracellular vesicles secreted by tumor cells promote the generation of suppressive monocytes. ImmunoHorizons 5, 647–658. 10.4049/immunohorizons.2000017 34404719

[B59] Martínez-Lorenzo M. J. Anel A. Gamen S. Monle n I. Lasierra P. Larrad L. (1999). Activated human T cells release bioactive Fas ligand and APO2 ligand in microvesicles. J. Immunol. Baltim. Md, 1950) 163, 1274–1281. 10415024

[B60] Mi Y. Guo N. Luan J. Cheng J. Hu Z. Jiang P. (2020). The emerging role of myeloid-derived suppressor cells in the glioma immune suppressive microenvironment. Front. Immunol. 11, 737–811. 10.3389/fimmu.2020.00737 32391020 PMC7193311

[B61] Miller K. D. Ostrom Q. T. Kruchko C. Patil N. Tihan T. Cioffi G. (2021). Brain and other central nervous system tumor statistics, 2021. CA A Cancer J. Clin. 71, 381–406. 10.3322/caac.21693 34427324

[B62] Mittelbrunn M. Gutiérrez-Vázquez C. Villarroya-Beltri C. González S. Sánchez-Cabo F. González M. Á. (2011). Unidirectional transfer of microRNA-loaded exosomes from T cells to antigen-presenting cells. Nat. Commun. 2, 282. 10.1038/ncomms1285 21505438 PMC3104548

[B63] Monleón I. Martínez-Lorenzo M. J. Monteagudo L. Lasierra P. Taulés M. Iturralde M. (2001). Differential secretion of Fas ligand- or APO2 ligand/TNF-related apoptosis-inducing ligand-carrying microvesicles during activation-induced death of human T cells. J. Immunol. 167, 6736–6744. 10.4049/jimmunol.167.12.6736 11739488

[B64] Moreno-Gonzalo O. Fernandez-Delgado I. Sanchez-Madrid F. (2018). Post-translational add-ons mark the path in exosomal protein sorting. Cell. Mol. Life Sci. 75, 1–19. 10.1007/s00018-017-2690-y 29080091 PMC11105655

[B65] Muntasell A. Berger A. C. Roche P. A. (2007). T cell-induced secretion of MHC class II–peptide complexes on B cell exosomes. EMBO J. 26, 4263–4272. 10.1038/sj.emboj.7601842 17805347 PMC2230838

[B66] Nolte-'t Hoen E. N. Wagenaar-Hilbers J. P. Peters P. J. Gadella B. M. van Eden W. Wauben M. H. (2004). Uptake of membrane molecules from T cells endows antigen-presenting cells with novel functional properties. Eur. J. Immunol. 34, 3115–3125. 10.1002/eji.200324711 15459903

[B67] Ohgaki H. Kleihues P. (2005). Population-based studies on incidence, survival rates, and genetic alterations in astrocytic and oligodendroglial gliomas. J. Neuropathol. Exp. Neurol. 64, 479–489. 10.1093/jnen/64.6.479 15977639

[B68] Ohgaki H. Kleihues P. (2007). Genetic pathways to primary and secondary glioblastoma. Am. J. Pathol. 170, 1445–1453. 10.2353/ajpath.2007.070011 17456751 PMC1854940

[B69] Ohgaki H. Kleihues P. (2013). The definition of primary and secondary glioblastoma. Clin. Cancer Res. 19, 764–772. 10.1158/1078-0432.CCR-12-3002 23209033

[B70] Okoye I. S. Coomes S. M. Pelly V. S. Czieso S. Papayannopoulos V. Tolmachova T. (2014). MicroRNA-containing T-regulatory-cell-derived exosomes suppress pathogenic T helper 1 cells. Immunity 41, 503–103. 10.1016/j.immuni.2014.08.008 28903020 PMC5640441

[B71] Ostrom Q. T. Cioffi G. Waite K. Kruchko C. Barnholtz-Sloan J. S. (2021). CBTRUS statistical report: Primary brain and other central nervous system tumors diagnosed in the United States in 2014-2018. Neuro. Oncol. 23, III1–III105. 10.1093/neuonc/noab200 34608945 PMC8491279

[B72] Oushy S. Hellwinkel J. E. Wang M. Nguyen G. J. Gunaydin D. Harland T. A. (2018). Glioblastoma multiforme-derived extracellular vesicles drive normal astrocytes towards a tumour-enhancing phenotype. Philos. Trans. R. Soc. Lond. B Biol. Sci. 373, 20160477. 10.1098/rstb.2016.0477 29158308 PMC5717433

[B73] Papadopoulos Z. Herz J. Kipnis J. (2020). Meningeal lymphatics: From anatomy to central nervous system immune surveillance. J. Immunol. 204, 286–293. 10.4049/jimmunol.1900838 31907271 PMC7061974

[B74] Pavlyukov M. S. Yu H. Bastola S. Minata M. Shender V. O. Lee Y. (2018). Apoptotic cell-derived extracellular vesicles promote malignancy of glioblastoma via intercellular transfer of splicing factors. Cancer Cell 34, 119–135. 10.1016/j.ccell.2018.05.012 29937354 PMC6048596

[B75] Pende D. Cantoni C. Rivera P. Vitale M. Castriconi R. Marcenaro S. (2001). Role of NKG2D in tumor cell lysis mediated by human NK cells: Cooperation with natural cytotoxicity receptors and capability of recognizing tumors of nonepithelial origin. Eur. J. Immunol. 31, 1076–1086. 10.1002/1521-4141(200104)31:4<1076:aid-immu1076>3.0.co;2-y 11298332

[B76] Piper R. C. Dikic I. Lukacs G. L. (2014). Ubiquitin-dependent sorting in endocytosis. Cold Spring Harb. Perspect. Biol. 6, a016808. 10.1101/cshperspect.a016808 24384571 PMC3941215

[B77] Qian M. Wang S. Guo X. Wang J. Zhang Z. Qiu W. (2020). Hypoxic glioma-derived exosomes deliver microRNA-1246 to induce M2 macrophage polarization by targeting TERF2IP via the STAT3 and NF-κB pathways. Oncogene 39, 428–442. 10.1038/s41388-019-0996-y 31485019

[B78] Quail D. F. Joyce J. A. (2017). The microenvironmental landscape of brain tumors. Cancer Cell 31, 326–341. 10.1016/j.ccell.2017.02.009 28292436 PMC5424263

[B79] Ramakrishnan V. Xu B. Akers J. Nguyen T. Ma J. Dhawan S. (2020). Radiation-induced extracellular vesicle (EV) release of miR-603 promotes IGF1-mediated stem cell state in glioblastomas. EBioMedicine 55, 102736. 10.1016/j.ebiom.2020.102736 32361246 PMC7195524

[B80] Raposo G. Nijman H. W. Stoorvogel W. Liejendekker R. Harding C. V. Melief C. J. (1996). B lymphocytes secrete antigen-presenting vesicles. J. Exp. Med. 183, 1161–1172. 10.1084/jem.183.3.1161 8642258 PMC2192324

[B81] Ricklefs F. Mineo M. Rooj A. K. Nakano I. Charest A. Weissleder R. (2016). Extracellular vesicles from high-grade glioma exchange diverse pro-oncogenic signals that maintain intratumoral heterogeneity. Cancer Res. 76, 2876–2881. 10.1158/0008-5472.CAN-15-3432 27013191 PMC4873326

[B82] Ricklefs F. L. Alayo Q. Krenzlin H. Mahmoud A. B. Speranza M. C. Nakashima H. (2018). Immune evasion mediated by PD-L1 on glioblastoma-derived extracellular vesicles. Sci. Adv. 4, eaar2766. 10.1126/sciadv.aar2766 29532035 PMC5842038

[B83] Salaud C. Alvarez-Arenas A. Geraldo F. Belmonte-Beitia J. Calvo G. F. Gratas C. (2020). Mitochondria transfer from tumor-activated stromal cells (TASC) to primary Glioblastoma cells. Biochem. Biophys. Res. Commun. 533, 139–147. 10.1016/j.bbrc.2020.08.101 32943183

[B84] Schuler P. J. Saze Z. Hong C. S. Muller L. Gillespie D. G. Cheng D. (2014). Human CD4+CD39+ regulatory T cells produce adenosine upon co-expression of surface CD73 or contact with CD73+ exosomes or CD73+ cells. Clin. Exp. Immunol. 177, 531–543. 10.1111/cei.12354 24749746 PMC4226604

[B85] Shao H. Chung J. Lee K. Balaj L. Min C. Carter B. S. (2015). Chip-based analysis of exosomal mRNA mediating drug resistance in glioblastoma. Nat. Commun. 6, 6999–9. 10.1038/ncomms7999 25959588 PMC4430127

[B86] Simon T. Pinioti S. Schellenberger P. Rajeeve V. Wendler F. Cutillas P. R. (2018). Shedding of bevacizumab in tumour cells-derived extracellular vesicles as a new therapeutic escape mechanism in glioblastoma. Mol. Cancer 17, 132. 10.1186/s12943-018-0878-x 30165850 PMC6117885

[B87] Skak K. Frederiksen K. S. Lundsgaard D. (2008). Interleukin-21 activates human natural killer cells and modulates their surface receptor expression. Immunology 123, 575–583. 10.1111/j.1365-2567.2007.02730.x 18005035 PMC2433320

[B88] Smyth L. A. Ratnasothy K. Tsang J. Y. Boardman D. Warley A. Lechler R. (2013). CD73 expression on extracellular vesicles derived from CD4+ CD25+ Foxp3+ T cells contributes to their regulatory function. Eur. J. Immunol. 43, 2430–2440. 10.1002/eji.201242909 23749427

[B89] Spinelli C. Montermini L. Meehan B. Brisson A. R. Tan S. Choi D. (2018). Molecular subtypes and differentiation programmes of glioma stem cells as determinants of extracellular vesicle profiles and endothelial cell-stimulating activities. J. Extracell. Vesicles 7, 1490144. 10.1080/20013078.2018.1490144 30034643 PMC6052423

[B90] Stensjøen A. L. Solheim O. Kvistad K. A. Håberg A. K. Salvesen Ø. Berntsen E. M. (2015). Growth dynamics of untreated glioblastomas *in vivo* . Neuro. Oncol. 17, 1402–1411. 10.1093/neuonc/nov029 25758748 PMC4578579

[B91] Thakur B. K. Zhang H. Becker A. Matei I. Huang Y. Costa-Silva B. (2014). Double-stranded DNA in exosomes: A novel biomarker in cancer detection. Cell Res. 24, 766–769. 10.1038/cr.2014.44 24710597 PMC4042169

[B92] Théry C. Witwer K. W. Aikawa E. Alcaraz M. J. Anderson J. D. Andriantsitohaina R. (2018). Minimal information for studies of extracellular vesicles 2018 (MISEV2018): a position statement of the international society for extracellular vesicles and update of the MISEV2014 guidelines. J. Extracell. Vesicles 7, 1535750. 10.1080/20013078.2018.1535750 30637094 PMC6322352

[B93] Tian Y. Wang Z. Wang Y. Yin B. Yuan J. Qiang B. (2020). Glioma-derived endothelial cells promote glioma cells migration via extracellular vesicles-mediated transfer of MYO1C. Biochem. Biophys. Res. Commun. 525, 155–161. 10.1016/j.bbrc.2020.02.017 32081419

[B94] Tomaszewski W. Sanchez-Perez L. Gajewski T. F. Sampson J. H. (2019). Brain tumor microenvironment and host state: Implications for immunotherapy. Clin. Cancer Res. 25, 4202–4210. 10.1158/1078-0432.CCR-18-1627 30804019 PMC6635001

[B95] Treps L. Edmond S. Harford-Wright E. Galan-Moya E. M. Schmitt A. Azzi S. (2016). Extracellular vesicle-transported Semaphorin3A promotes vascular permeability in glioblastoma. Oncogene 35, 2615–2623. 10.1038/onc.2015.317 26364614

[B96] Treps L. Perret R. Edmond S. Ricard D. Gavard J. (2017). Glioblastoma stem-like cells secrete the pro-angiogenic VEGF-A factor in extracellular vesicles. J. Extracell. Vesicles 6, 1359479. 10.1080/20013078.2017.1359479 28815003 PMC5549846

[B97] Tsutsui T. Kawahara H. Kimura R. Dong Y. Jiapaer S. Sabit H. (2020). Glioma-derived extracellular vesicles promote tumor progression by conveying WT1. Carcinogenesis 41, 1238–1245. 10.1093/carcin/bgaa052 32463428

[B98] Tung S. L. Boardman D. A. Sen M. Letizia M. Peng Q. Cianci N. (2018). Regulatory T cell-derived extracellular vesicles modify dendritic cell function. Sci. Rep. 8, 6065–6112. 10.1038/s41598-018-24531-8 29666503 PMC5904112

[B99] Urabe F. Kosaka N. Ito K. Kimura T. Egawa S. Ochiya T. (2020). Extracellular vesicles as biomarkers and therapeutic targets for cancer. Am. J. Physiol. Cell Physiol. 318, C29–C39. 10.1152/ajpcell.00280.2019 31693397

[B100] Vagner T. Spinelli C. Minciacchi V. R. Balaj L. Zandian M. Conley A. (2018). Large extracellular vesicles carry most of the tumour DNA circulating in prostate cancer patient plasma. J. Extracell. Vesicles 7, 1505403. 10.1080/20013078.2018.1505403 30108686 PMC6084494

[B101] Van Der Vos K. E. Abels E. R. Zhang X. Lai C. Carrizosa E. Oakley D. (2016). Directly visualized glioblastoma-derived extracellular vesicles transfer RNA to microglia/macrophages in the brain. Neuro. Oncol. 18, 58–69. 10.1093/neuonc/nov244 26433199 PMC4677420

[B102] Viel S. Marçais A. Guimaraes F. S. F. Loftus R. Rabilloud J. Grau M. (2016). TGF-β inhibits the activation and functions of NK cells by repressing the mTOR pathway. Sci. Signal. 9, ra19–14. 10.1126/scisignal.aad1884 26884601

[B103] Villarroya-Beltri C. Gutiérrez-Vázquez C. Sánchez-Cabo F. Pérez-Hernández D. Vázquez J. Martin-Cofreces N. (2013). Sumoylated hnRNPA2B1 controls the sorting of miRNAs into exosomes through binding to specific motifs. Nat. Commun. 4, 2980–3010. 10.1038/ncomms3980 24356509 PMC3905700

[B104] Wagner K. D. Cherfils-Vicini J. Hosen N. Hohenstein P. Gilson E. Hastie N. D. (2014). The Wilms tumour suppressor Wt1 is a major regulator of tumour angiogenesis and progression. Nat. Commun. 5, 5852. 10.1038/ncomms6852 25510679

[B105] Walker L. S. K. Sansom D. M. (2011). The emerging role of CTLA4 as a cell-extrinsic regulator of T cell responses. Nat. Rev. Immunol. 11, 852–863. 10.1038/nri3108 22116087

[B106] Yang J. Dang G. Lü S. Liu H. Ma X. Han L. (2019). T‐cell–derived extracellular vesicles regulate B‐cell IgG production via pyruvate kinase muscle isozyme 2. FASEB J. 33, 12780–12799. 10.1096/fj.201900863R 31480861 PMC6902684

[B107] Yekula A. Minciacchi V. R. Morello M. Shao H. Park Y. Zhang X. (2020). Large and small extracellular vesicles released by glioma cells *in vitro* and *in vivo* . J. Extracell. Vesicles 9, 1689784. 10.1080/20013078.2019.1689784 31839905 PMC6896449

[B108] Yu M. W. Quail D. F. (2021). Immunotherapy for glioblastoma: Current progress and challenges. Front. Immunol. 12, 676301. 10.3389/fimmu.2021.676301 34054867 PMC8158294

[B109] Yue X. Lan F. Xia T. (2019). Hypoxic glioma cell-secreted exosomal miR-301a activates wnt/β-catenin signaling and promotes radiation resistance by targeting TCEAL7. Mol. Ther. 27, 1939–1949. 10.1016/j.ymthe.2019.07.011 31402274 PMC6838947

[B110] Zhang F. Li R. Yang Y. Shi C. Shen Y. Lu C. (2019). Specific decrease in B-Cell-Derived extracellular vesicles enhances post-chemotherapeutic CD8 + T cell responses. Immunity 50, 738–750. 10.1016/j.immuni.2019.01.010 30770248

[B111] Zhang Z. Xu J. Chen Z. Wang H. Xue H. Yang C. (2020). Transfer of MicroRNA via macrophage-derived extracellular vesicles promotes proneural-to-mesenchymal transition in glioma stem cells. Cancer Immunol. Res. 8, 966–981. 10.1158/2326-6066.CIR-19-0759 32350000

[B112] Zuccato E. Blott E. J. Holt O. Sigismund S. Shaw M. Bossi G. (2007). Sorting of Fas ligand to secretory lysosomes is regulated by mono-ubiquitylation and phosphorylation. J. Cell Sci. 120, 191–199. 10.1242/jcs.03315 17164290

